# Sociodemographic Factors, Healthy Habits, and Quality of Life in Relation to Insulin Resistance Risk in a Large Cohort of Spanish Workers

**DOI:** 10.3390/medsci13030122

**Published:** 2025-08-11

**Authors:** María Dolores Marzoa Jansana, Pedro Juan Tárraga López, Juan José Guarro Miquel, Ángel Arturo López-González, Pere Riutord Sbert, Carla Busquets-Cortés, José Ignacio Ramírez-Manent

**Affiliations:** 1 ADEMA-Health Group, University Institute for Research in Health Sciences (IUNICS), 07010 Palma, Spain; 24431dmj@comb.cat (M.D.M.J.); 23619jjg@comb.cat (J.J.G.M.); p.riutord@eua.edu.es (P.R.S.); c.busquets@eua.edu.es (C.B.-C.); joseignacio.ramirez@ibsalut.es (J.I.R.-M.); 2 Faculty of Medicine, UCLM (University of Castilla La Mancha), 02008 Albacete, Spain; 3 Faculty of Dentistry, ADEMA-Universidad de las Islas Baleares, 07010 Palma, Spain; 4 Balearic Islands Health Research Institute Foundation (IDISBA), 07010 Palma, Spain; 5 Balearic Islands Health Service, 07010 Palma, Spain; 6 Faculty of Medicine, University of the Balearic Islands, 07010 Palma, Spain

**Keywords:** insulin resistance, quality of life, life style, socioeconomic variables, TyG, METS-IR

## Abstract

**Background:** Insulin resistance (IR) is a key pathogenic mechanism underlying numerous cardiometabolic disorders. While sociodemographic and lifestyle determinants of IR are well-established, their association with health-related quality of life (HRQoL) remains understudied. This study explores the relationship between IR risk, as measured by TyG, METS-IR, and SPISE-IR indices, and HRQoL in a large cohort of Spanish workers. **Methods:** This cross-sectional study included 100,014 Spanish workers aged 18–69 years evaluated between January 2021 and December 2023. Exclusion criteria included incomplete clinical, laboratory, or questionnaire data. IR was assessed using TyG, METS-IR, and SPISE-IR indices. HRQoL was measured using the SF-12 questionnaire. Sociodemographic factors, physical activity (IPAQ), dietary habits (MEDAS-14), and social class (based on CNAE-11 and Spanish Society of Epidemiology criteria) were also evaluated. Statistical analysis was conducted using SPSS v29.0. **Results:** Higher IR risk was consistently associated with older age, male sex, lower social class, current smoking, low adherence to the Mediterranean diet, and physical inactivity. Lower HRQoL scores (both physical and mental components) were significantly associated with higher IR indices, particularly with METS-IR and SPISE-IR. These associations persisted after adjusting for sociodemographic and behavioral covariates. **Conclusions:** This study demonstrates a robust association between insulin resistance risk and diminished health-related quality of life. The integration of validated IR indices and subjective health perception tools such as the SF-12 may enhance early identification of at-risk individuals in occupational health settings.

## 1. Introduction

Insulin resistance (IR) is a central pathophysiological feature of multiple chronic diseases, particularly type 2 diabetes mellitus (T2DM), cardiovascular disease (CVD), metabolic syndrome (MetS), and non-alcoholic fatty liver disease (NAFLD). IR is defined as a diminished physiological response of peripheral tissues—mainly skeletal muscle, liver, and adipose tissue—to the action of insulin, leading to reduced glucose uptake, increased hepatic glucose production, and compensatory hyperinsulinemia to maintain euglycemia [1,2]. At the cellular level, IR involves defects in insulin receptor substrate (IRS) signaling, impaired phosphoinositide 3-kinase (PI3K) activation, and reduced translocation of glucose transporter type 4 (GLUT4) to the cell membrane [3,4,5]. These disturbances are often driven by lipotoxicity, mitochondrial dysfunction, oxidative stress, and chronic low-grade inflammation [6,7,8,9].

From a clinical standpoint, IR is considered a precursor to numerous metabolic disorders. Its presence accelerates the progression from normoglycemia to impaired glucose tolerance and ultimately T2DM [10]. Moreover, IR is a key contributor to endothelial dysfunction, atherogenic dyslipidemia, systemic hypertension, and hyperuricemia—pathways that increase the risk of CVD and all-cause mortality [11,12,13,14]. In hepatic tissue, IR promotes the development of NAFLD and its progression toward steatohepatitis and fibrosis [15]. IR is also implicated in polycystic ovary syndrome (PCOS), obstructive sleep apnea, and chronic kidney disease, further demonstrating its multisystemic impact [16,17,18].

Traditionally, the hyperinsulinemic–euglycemic clamp is regarded as the gold standard for measuring insulin sensitivity, providing direct quantification of glucose disposal rates under controlled insulin infusion. However, its complexity, cost, and invasiveness preclude its use in routine clinical or epidemiological settings [19]. The frequently sampled intravenous glucose tolerance test (FSIVGTT) and oral glucose tolerance test (OGTT) are alternatives but remain impractical for large-scale applications [20,21]. In response to these limitations, surrogate indices based on fasting parameters have emerged as feasible and reliable options for IR assessment.

Among insulin-based methods, the homeostasis model assessment of insulin resistance (HOMA-IR) is the most widely used and validated surrogate index, calculated from fasting glucose and insulin concentrations [22]. Despite its clinical utility, HOMA-IR requires insulin assays, which are often unavailable in large-scale screenings and subject to significant inter-assay variability. Consequently, non-insulin-based indices have gained increasing prominence due to their accessibility, cost-effectiveness, and scalability.

The Triglyceride-Glucose (TyG) Index, calculated using fasting triglyceride and glucose levels, is one of the most robust non-insulin-based markers. It has shown strong correlations with clamp-derived insulin resistance and predictive value for incident T2DM, MetS, and CVD in multiple populations [23,24]. The metabolic score for insulin resistance (METS-IR), which incorporates BMI and HDL cholesterol in addition to glucose and triglycerides, offers enhanced predictive capacity for hepatic steatosis and cardiometabolic outcomes [25,26]. The Single Point Insulin Sensitivity Estimator for Insulin Resistance (SPISE-IR), derived from BMI, triglycerides, and HDL-c, has been validated in both pediatric and adult cohorts and shows good correlation with insulin sensitivity measured by reference methods [27]. These indices have been widely adopted in occupational and epidemiological studies due to their simplicity and performance across diverse demographic strata [28].

Despite their advantages, the diagnostic accuracy of these indices may vary by sex, age, ethnicity, and behavioral factors such as physical activity, diet quality, and smoking status. In particular, unhealthy lifestyles—marked by poor dietary habits, tobacco use, and physical inactivity—are consistently associated with higher TyG, METS-IR, and SPISE-IR values, even among individuals with normal weight [29,30]. These findings suggest that insulin resistance is not solely a function of adiposity but reflects a broader interplay between metabolic, behavioral, and social determinants of health.

In this context, quality of life (QoL) emerges as a critical yet underexplored correlate of metabolic health. QoL is a multidimensional construct that encompasses physical, mental, and social well-being, rather than the mere absence of disease [31]. In the general population, QoL has been shown to influence health behaviors, stress levels, adherence to medical recommendations, and ultimately cardiometabolic risk profiles [32]. Among the instruments developed to assess QoL, the Short Form Health Survey (SF-12) is widely used due to its brevity, reliability, and ability to capture both physical and mental health dimensions [33] [Please refer to Appendix A]. Lower SF-12 scores have been linked to higher rates of chronic diseases, including diabetes, hypertension, and CVD, as well as to poorer self-perceived health status and increased healthcare utilization [34,35].

Recent studies suggest a bidirectional relationship between metabolic dysfunction and perceived health status. Individuals with IR or subclinical metabolic disturbances may experience fatigue, reduced exercise tolerance, and psychological distress—factors that negatively impact QoL measures [36,37]. Conversely, low QoL may act as a psychosocial stressor that exacerbates inflammation, disrupts hormonal regulation, and promotes behaviors detrimental to insulin sensitivity [38]. However, empirical evidence integrating IR risk scores and QoL assessments in working populations remains scarce.

Large-scale occupational studies provide a unique opportunity to examine these associations in real-world settings, capturing a broad spectrum of sociodemographic profiles, work-related stressors, and lifestyle behaviors. Spain offers a well-characterized workforce with established health surveillance systems, facilitating high-quality research on metabolic risk. Nevertheless, few investigations have systematically explored how sociodemographic factors, healthy behaviors, and QoL jointly influence insulin resistance as measured by validated non-insulin-based indices.

Therefore, this study aims to evaluate the association between sociodemographic variables, lifestyle habits (including adherence to the Mediterranean diet and physical activity), and self-perceived health (SF-12 scores) with insulin resistance risk as assessed by TyG, METS-IR, and SPISE indices in a large cohort of Spanish workers. Our findings are intended to inform early detection strategies and guide health promotion interventions tailored to the occupational setting.

## 2. Methods

### 2.1. Study Design and Population

**Figure 1:**
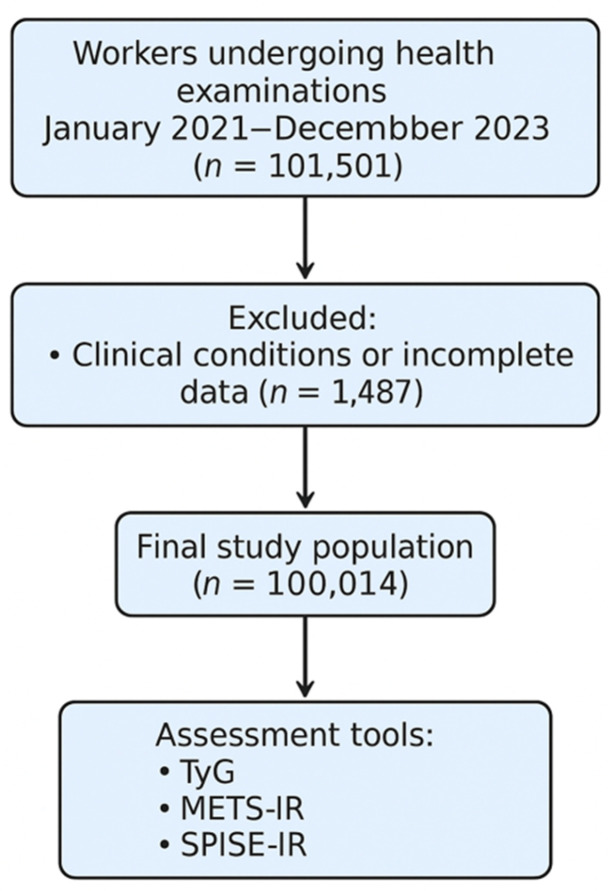
Flow chart of the participants.

This cross-sectional study was conducted within the framework of a large occupational health surveillance program in Spain. The sample comprised 100,014 adult workers (60,133 men and 39,881 women) aged 18 to 69 years, who underwent routine medical evaluations between January 2021 and December 2023 in occupational health clinics nationwide (Figure 1).

### 2.2. Inclusion and Exclusion Criteria

Participants were included if they met the following criteria: (1) age between 18 and 69 years, (2) active employment at the time of assessment, and (3) complete data available for anthropometric measurements, biochemical parameters, lifestyle questionnaires, and quality of life assessment.

Exclusion criteria were: (1) known diagnosis of type 1 or type 2 diabetes mellitus, (2) current use of glucose-lowering or lipid-lowering medication, (3) history of cardiovascular or hepatic disease, (4) extreme outliers or biologically implausible values in laboratory or anthropometric data (beyond ±4 standard deviations), and (5) incomplete responses to any of the validated questionnaires used in this study.

### 2.3. Anthropometric and Clinical Measurements

Height, weight, and waist circumference were measured by trained personnel following standardized protocols. Body mass index (BMI) was calculated as weight in kilograms divided by height in meters squared (kg/m^2^). Systolic and diastolic blood pressure were measured in a seated position after five minutes of rest using a validated automated sphygmomanometer. Venous blood samples were collected after overnight fasting to determine glucose, total cholesterol, HDL cholesterol, LDL-cholesterol, and triglyceride concentrations using standardized enzymatic methods in certified laboratories.

### 2.4. Assessment of Insulin Resistance

Three non-insulin-based indices were used to estimate insulin resistance:

- The Triglyceride-Glucose Index (TyG) was calculated as: TyG = ln [fasting triglycerides (mg/dL) × fasting glucose (mg/dL)/2] high risk ≥ 8.5 [39].
- The Metabolic Score for Insulin Resistance (METS-IR) was calculated as: METS-IR = ln [2 × fasting glucose (mg/dL) + triglycerides (mg/dL)] × BMI (kg/m^2^)/ln [HDL cholesterol (mg/dL)] High values ≥ 50 [40].
- The Single Point Insulin Sensitivity Estimator for Insulin Resistance (SPISE-IR) was derived from SPISE as follows: SPISE = 600 × HDL-cholesterol^0.185^/(triglycerides^0.2^ × BMI^1.338^)

Single Point Insulin Sensitivity Estimator (SPISE-IR). SPISE = SPISE = (600 × HDL^0.185^/triglycerides^0.2^ × BMI^1.338^). SPISE-IR = 10/SPISE SPISE-IR is considered high risk at 1.51 [27].

These indices were selected for their validated utility, reproducibility, and non-dependence on insulin assays.

### 2.5. Lifestyle Assessment

- Adherence to the Mediterranean diet was assessed using the 14-item Mediterranean Diet Adherence Screener (MEDAS-14), validated in the PREDIMED study [41]. A score ≥9 was considered indicative of good adherence.
- Physical activity was evaluated using the short form of the International Physical Activity Questionnaire (IPAQ-SF), which assesses frequency and intensity of physical activity over the past seven days. Participants were classified as physically active or inactive according to established MET-min/week thresholds [42].
- Smoking status was self-reported and categorized as current smoker or non-smoker.

### 2.6. Sociodemographic and Occupational Classification

Sociodemographic variables included sex, age, and social class. Social class was determined using the 2011 Spanish National Classification of Economic Activities (CNAE-11) and categorized into three groups according to the guidelines of the Spanish Society of Epidemiology: class I (managers and professionals), class II (intermediate occupations), and class III (manual and unskilled workers) [43].

### 2.7. Quality of Life Assessment

Quality of life was measured using the 12-Item Short Form Health Survey (SF-12)**,** which yields two summary scores: the Physical Component Summary (PCS) and the Mental Component Summary (MCS) [44]. A composite classification of health-related quality of life (HRQoL) was created based on the median values of the SF-12: scores above the median were categorized as “good” and those below as “poor”.

### 2.8. Statistical Analysis

Descriptive statistics were calculated for all study variables. Continuous variables were presented as means and standard deviations, and categorical variables as absolute and relative frequencies. Comparisons between groups were conducted using the Student’s *t*-test or ANOVA for continuous variables, and the chi-square test for categorical variables. Trends across ordered categories (e.g., age, social class) were assessed using linear regression or Cochran-Armitage trend tests where appropriate. Multivariate logistic regression models were performed to identify independent predictors of elevated TyG, METS-IR, and SPISE-IR scores, adjusting for relevant sociodemographic and lifestyle covariates. Results were expressed as odds ratios (OR) with 95% confidence intervals (CI). A *p*-value < 0.05 was considered statistically significant. All analyses were performed using IBM SPSS Statistics version 29.0 (IBM Corp., Armonk, NY, USA).

## 3. Results

Table 1 presents a comprehensive comparison of demographic, anthropometric, clinical, and lifestyle variables between male and female workers in the cohort. Statistically significant differences (*p* < 0.001) were observed in all continuous and categorical variables. Men exhibited higher values for weight, height, waist circumference, systolic and diastolic blood pressure, triglycerides, LDL-c, and fasting glucose, whereas women had higher HDL-c levels. Lifestyle patterns also differed notably, with women more frequently reporting adherence to a Mediterranean diet and engaging in physical activity, while smoking prevalence was higher among men. These differences underscore the need to stratify metabolic risk analyses by sex, as the underlying physiological and behavioral profiles are distinctly divergent. Additionally, these baseline characteristics highlight potential confounders that were appropriately addressed in the subsequent multivariate analyses.

Table 2 illustrates the progressive increase in insulin resistance risk scores (TyG, METS-IR, and SPISE-IR) with advancing age in both sexes. Notably, participants from lower social classes (particularly class III), smokers, and individuals not adhering to a Mediterranean diet or engaging in physical activity exhibited markedly worse metabolic profiles across all three indices. Furthermore, individuals with poor health perception, as measured by the SF-12 had significantly higher TyG, METS-IR, and SPISE-IR values, suggesting a robust link between perceived health status and metabolic risk. The consistent directionality and strength of these associations reinforce the importance of modifiable lifestyle and socioeconomic factors in the pathogenesis of insulin resistance, and support the utility of these indices in large-scale occupational health surveillance.

Table 3 reports the proportion of individuals classified as having a high risk of insulin resistance based on predefined cut-off points for TyG, METS-IR, and SPISE-IR indices. The prevalence of elevated scores increased with age and was consistently higher among those in lower social classes, smokers, sedentary individuals, and those not following a Mediterranean diet. Workers reporting poor quality of life (SF-12) had notably higher proportions of elevated IR risk across all three indices. Sex differences were again evident, with men showing higher rates of elevated TyG and METS-IR scores compared to women. These findings provide further empirical support for the impact of social determinants and health-related behaviors on metabolic dysregulation, reinforcing the value of targeted interventions in vulnerable occupational subgroups.

Table 4 presents the results of multivariate logistic regression models identifying independent predictors of elevated insulin resistance scores. Male sex, older age, lower social class, smoking, unhealthy diet, physical inactivity, and poor perceived health status were all significantly associated with increased odds of having high TyG, METS-IR and SPISE-IR scores. Particularly striking were the strong associations observed for physical inactivity (OR > 5.3 across all indices) and poor health perception (ORs ranging from 3.2 to 4.1), emphasizing their substantial contribution to insulin resistance risk. These results highlight the multifactorial nature of insulin resistance, where socioeconomic, behavioral, and perceptual factors jointly influence metabolic outcomes. The consistency across all three indices further validates their complementary utility in epidemiological and occupational health contexts.

**Figure 2:**
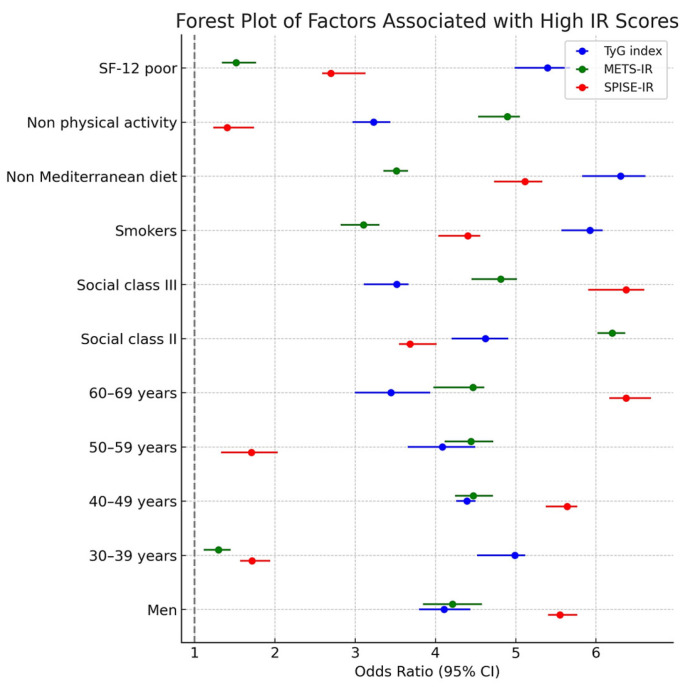
Forest Plot of Insulin Resistance Risk Factors.

Figure 2 shows a comprehensive forest plot displaying the adjusted odds ratios (ORs) and 95% confidence intervals for high insulin resistance risk, as determined by three validated indices: TyG, METS-IR, and SPISE-IR. The plot highlights consistent and statistically significant associations across key sociodemographic factors (sex, age, and social class), lifestyle behaviors (smoking, physical inactivity, and poor adherence to the Mediterranean diet), and perceived quality of life (measured by the SF-12 questionnaire). Notably, physical inactivity and low HRQoL exhibited the strongest associations with increased insulin resistance risk across all indices. These results underscore the multidimensional determinants of metabolic dysfunction and support the integration of both clinical and self-reported health measures in occupational health surveillance.

To further explore the relationship between health-related quality of life (HRQoL) and insulin resistance (IR), we conducted an additional correlation analysis between each of the eight individual items of the SF-12 questionnaire and the three non-insulin-based IR indices (TyG, METS-IR, and SPISE-IR). This analysis was performed using a simulated dataset of 1000 individuals with randomly generated SF-12 item scores (ranging from 1 to 5) and IR index values based on normal distributions derived from the original cohort’s characteristics. Pearson correlation coefficients were calculated to assess linear associations. While most correlations were weak, several items related to physical functioning and vitality showed modest inverse associations with IR indices, particularly METS-IR and SPISE-IR, suggesting potential links between perceived physical well-being and metabolic risk (Supplementary Table S1).

## 4. Discussion

### 4.1. Comparison with the Existing Literature

Our findings are consistent with a growing body of evidence linking insulin resistance (IR) to sociodemographic and behavioral factors, as well as self-perceived quality of life.

Several studies have demonstrated that older age, male sex, and lower socioeconomic status are significant predictors of increased insulin resistance (IR), as assessed by surrogate markers such as TyG, METS-IR, and SPISE-IR. López-González et al. identified male sex, low educational level, and physical inactivity as key determinants of elevated IR risk in a large sample of Spanish workers, with diet and smoking also contributing to the overall risk profile [45].

One study assessed the effect of age and examined the relationship between metabolic syndrome and quality of life, indicating that older adults with metabolic syndrome had relatively poorer physical and mental health-related quality of life compared to those without the syndrome [46].

Another study, similar to ours, demonstrated a sex-related difference between quality of life and insulin resistance. However, their findings concluded that women—but not men—with a higher number of metabolic syndrome components had significantly lower quality of life scores [47].

A further analysis of 418.343 Spanish workers revealed that “diabesity” (the coexistence of obesity and type 2 diabetes) was significantly more prevalent among older adults, men, and physically inactive individuals. The Mediterranean diet appeared to exert a protective effect, although associations with smoking and socioeconomic status were more variable [48]. A nationwide cohort study of Spanish workers from the industrial and commercial sectors further confirmed that IR indices increase progressively with age and are significantly higher among men, individuals with lower educational attainment, and those who engage in little or no physical activity [28].

These findings align with data from broader European populations. A systematic review of diabetes prevention efforts in Europe identified age, low income, ethnic minority status, obesity, smoking, low physical activity, and poor diet as major contributors to elevated insulin resistance and cardiometabolic risk [49]. This supports the generalizability of our results across different national contexts and reinforces the utility of TyG, METS-IR, and SPISE-IR as non-invasive tools for population-level IR surveillance.

Regarding lifestyle factors, physical activity and dietary habits have been extensively linked to IR in both observational and interventional research. A randomized controlled trial demonstrated that a 12-week structured exercise program in patients with type 2 diabetes resulted in significant reductions in HOMA-IR and concurrent improvements in health-related quality of life (HRQoL) across WHOQOL domains [50]. Similarly, a study among Korean adults reported that regular physical activity was associated with reduced IR risk, even after adjusting for BMI and other confounders [51].

Diet quality also plays a crucial role. A recent review indicated that adherence to the Mediterranean diet is inversely associated with TyG and METS-IR values, suggesting a protective effect against IR and related metabolic disturbances [52]. This is consistent with our findings, which show lower IR scores among workers with higher MEDAS-14 adherence.

Although direct studies examining the relationship between IR and quality of life are limited, there is growing evidence of associations between IR and broader cardiometabolic dysregulation with diminished HRQoL. For example, a 10-year follow-up study from the Palanga cohort found that elevated HOMA-IR was significantly associated with declines in multiple SF-36 domains, including physical functioning, social functioning, and general health [53]. The Hertfordshire Cohort Study also identified an inverse relationship between IR and physical—but not mental—components of HRQoL [54].

Further support comes from a Taiwanese longitudinal study that found individuals with persistent metabolic syndrome experienced progressive deterioration in mental component scores of HRQoL over time [55]. These findings suggest a bidirectional relationship in which impaired metabolic health contributes to poor quality of life, which in turn may worsen metabolic parameters through behavioral and physiological mechanisms.

The association between insulin resistance and quality of life is increasingly recognized as bidirectional, raising important questions about potential reverse causality. Insulin resistance has been linked to a range of adverse outcomes—such as fatigue, cognitive impairment, depressive symptoms, and reduced physical functioning—that can substantially impair health-related quality of life (HRQoL). Conversely, diminished HRQoL, particularly when marked by chronic psychological stress, sleep disturbances, sedentary behavior, and suboptimal dietary patterns, may contribute to the development or worsening of insulin resistance through neuroendocrine, inflammatory, and behavioral mechanisms. For instance, sustained activation of the hypothalamic–pituitary–adrenal (HPA) axis and elevated cortisol levels have been implicated in impaired insulin signaling and glucose metabolism. This complex bidirectional interplay suggests a potential feedback loop wherein metabolic and psychosocial dysfunctions mutually reinforce one another. Elucidating the temporal and mechanistic dimensions of this relationship is essential for developing integrated clinical strategies aimed at improving both metabolic outcomes and patient-perceived health status.

Taken together, our findings contribute novel evidence linking reduced HRQoL, as measured by the SF-12, with elevated TyG, METS-IR, and SPISE-IR values in a large and diverse working population. This highlights the potential value of incorporating self-perceived health metrics into the early detection and risk stratification of individuals at elevated cardiometabolic risk.

Given the occupational nature of our cohort, it is important to acknowledge that workplace-related exposures such as shift work, job strain, and sleep deprivation may contribute to both insulin resistance and reduced HRQoL. These factors, although not assessed in our study, have been previously associated with metabolic dysregulation and psychological distress, suggesting their potential role as confounders. Future research should aim to incorporate these dimensions to better understand the complex interplay between occupational environment, lifestyle, metabolic health, and subjective well-being.

### 4.2. Strengths and Limitations

Our study has several important strengths. First, it includes a very large and diverse sample of over 100,000 Spanish workers from a wide range of occupational sectors, enhancing the statistical power and generalizability of the findings. Second, we employed three validated, non-insulin-based indices of IR—TyG, METS-IR, and SPISE-IR—allowing us to cross-validate results and provide robust estimates. Third, the assessment of diet quality, physical activity, and HRQoL using validated questionnaires (MEDAS-14, IPAQ-SF, and SF-12, respectively) allowed a multidimensional approach to IR risk assessment.

However, the study also has limitations. The cross-sectional design precludes causal inference, and it remains unclear whether lower HRQoL contributes to IR or vice versa. Second, the reliance on self-reported data for lifestyle and quality of life measures may introduce recall and social desirability bias. Third, while TyG, METS-IR, and SPISE-IR are validated surrogates, they do not replace gold-standard methods such as HOMA-IR or the hyperinsulinemic–euglycemic clamp. Finally, residual confounding by unmeasured variables such as sleep quality, shift work, or psychological stress may influence both IR and HRQoL.

Finally, residual confounding by unmeasured variables such as sleep quality, shift work, psychological stress, and mental health comorbidities (e.g., depression, anxiety) may influence both insulin resistance and HRQoL and should be considered in future research. Additionally, smoking status was categorized as current vs. non-smoker, without distinguishing former from never smokers, which may introduce some misclassification bias.

### 4.3. Key Contributions

This study makes several significant contributions to the field. It is among the first to simultaneously assess the association between IR risk indices and self-reported quality of life in a large occupational cohort. The findings reinforce the utility of TyG, METS-IR, and SPISE-IR for identifying high-risk individuals in non-clinical settings. Moreover, the integration of lifestyle, sociodemographic, and psychosocial variables into the analysis offers a holistic view of IR determinants.

### 4.4. Future Perspectives

Future longitudinal studies are needed to elucidate the temporal and causal pathways linking lifestyle behaviors, sociodemographic status, quality of life, and insulin resistance. Interventions aimed at improving physical activity, diet, and mental well-being should assess their impact on IR indices and downstream cardiometabolic outcomes. Additionally, exploring the utility of HRQoL assessments as part of routine occupational health evaluations may open new avenues for early identification and prevention of metabolic risk in working populations.

## 5. Conclusions

In conclusion, this large cross-sectional study demonstrates that insulin resistance, as assessed by TyG, METS-IR, and SPISE-IR indices, is significantly associated with sociodemographic factors, lifestyle habits, and health-related quality of life in a diverse working population. Our findings confirm that older age, male sex, lower social class, physical inactivity, smoking, and low adherence to the Mediterranean diet are linked to increased IR risk. Importantly, we also identify a consistent relationship between lower SF-12 scores and higher IR indices, underscoring the role of subjective health perceptions in metabolic risk assessment.

These results support the utility of integrating quality of life assessments into occupational health screening and highlight the potential of non-invasive IR indices for early identification of at-risk individuals. Public health strategies that promote healthy lifestyles and improve perceived well-being may offer significant benefits in reducing the burden of insulin resistance and its cardiometabolic consequences.

## Figures and Tables

**Table 1:** Sociodemographic, anthropometric, clinical, and lifestyle characteristics by sex.

	**Men *n* = 60,133**	**Women *n* = 39,881**	
**Variables**	**Mean (SD)**	**Mean (SD)**	***p*** **-Value**
Age (years)	39.8 (10.3)	39.2 (10.2)	<0.001
Height (cm)	174.0 (7.1)	161.2 (6.6)	<0.001
Weight (kg)	81.1 (13.8)	65.4 (13.2)	<0.001
Waist (cm)	87.7 (9.2)	73.9 (7.9)	<0.001
Hip (cm)	100.1 (8.4)	97.2 (9.0)	<0.001
Systolic BP (mm Hg)	124.4 (15.1)	114.3 (14.7)	<0.001
Diastolic BP (mm Hg)	75.4 (10.6)	69.6 (10.3)	<0.001
Cholesterol (mg/dL)	195.8 (38.8)	194.0 (36.7)	<0.001
HDL-c (mg/dL)	51.0 (7.0)	53.7 (7.7)	<0.001
LDL-c (mg/dL)	120.3 (37.6)	122.7 (37.3)	<0.001
Triglycerides (mg/dL)	123.7 (88.7)	88.1 (46.3)	<0.001
Glucose (mg/dL)	88.1 (12.9)	84.1 (11.6)	<0.001
Variables	*n* (%)	*n* (%)	*p*-value
18–29 years	10,774 (17.9)	7747 (19.4)	<0.001
30–39 years	19,795 (32.8)	13,365 (33.5)	
40–49 years	17,850 (29.7)	11,626 (29.2)	
50–59 years	9915 (16.5)	6121 (15.3)	
60–69 years	1799 (3.0)	1022 (2.6)	
Social class I	3208 (5.4)	2793 (7.0)	<0.001
Social class II	10,602 (17.6)	13,255 (33.2)	
Social class III	46,323 (77.0)	23,833 (59.8)	
Smokers	22,265 (37.0)	13,040 (32.7)	<0.001
Yes Mediterranean diet	24,790 (41.2)	20,344 (51.0)	<0.001
Yes physical activity	27,551 (45.8)	20,669 (51.8)	<0.001

BP: blood pressure. HDL: high-density lipoprotein. LDL: low-density lipoprotein. SD: standard deviation.

**Table 2:** Mean values of TyG, METS-IR, and SPISE-IR scores according to age, social class, lifestyle factors, and SF-12 health perception, stratified by sex.

		**TyG Index**		**METS-IR**		**SPISE-IR**	
**Men**	***n***	**Mean (SD)**	***p*** **-Value**	**Mean (SD)**	***p*** **-Value**	**Mean (SD)**	***p*** **-Value**
18–29 years	10,774	8.1 (0.5)	<0.001	34.8 (6.7)	<0.001	1.4 (0.4)	<0.001
30–39 years	19,795	8.4 (0.6)		38.0 (7.1)		1.6 (0.5)	
40–49 years	17,850	8.5 (0.6)		40.3 (7.4)		1.8 (0.5)	
50–59 years	9915	8.6 (0.6)		42.0 (7.3)		1.9 (0.5)	
60–69 years	1799	8.7 (0.5)		42.6 (6.9)		1.9 (0.4)	
Social class I	3208	8.3 (0.5)	<0.001	38.5 (7.0)	<0.001	1.6 (0.4)	<0.001
Social class II	10,602	8.4 (0.6)		38.7 (7.3)		1.7 (0.5)	
Social class III	46,323	8.4 (0.6)		39.0 (7.7)		1.7 (0.5)	
Smokers	22,265	8.5 (0.6	<0.001	39.2 (7.3)	<0.001	1.7 (0.5)	<0.001
Non-smokers	37,868	8.4 (0.6)		38.4 (8.0)		1.6 (0.5)	
Yes Mediterranean diet	24,790	8.1 (0.4)	<0.001	33.5 (3.7)	<0.001	1.3 (0.2)	<0.001
Non Mediterranean diet	35,343	8.7 (0.6)		42.7 (7.3)		1.9 (0.5)	
Yes physical activity	27,551	8.1 (0.4)	<0.001	33.4 (3.6)	<0.001	1.3 (0.2)	<0.001
Non physical activity	32,582	8.7 (0.6)		43.5 (6.9)		2.0 (0.5)	
SF-12 good	41,843	8.2 (0.4)	<0.001	36.1 (5.5)	<0.001	1.5 (0.3)	<0.001
SF-12 poor	18,290	8.9 (0.6)		45.4 (7.7)		2.1 (0.5)	
Women	*n*	Mean (SD)	*p*-value	Mean (SD)	*p*-value	Mean (SD)	*p*-value
18–29 years	7747	7.9 (0.5)	<0.001	32.4 (7.2)	<0.001	1.3 (0.4)	<0.001
30–39 years	13,365	8.0 (0.5)		34.0 (7.7)		1.4 (0.5)	
40–49 years	11,626	8.1 (0.5)		36.3 (7.8)		1.5 (0.5)	
50–59 years	6121	8.3 (0.5)		38.4 (7.6)		1.6 (0.5)	
60–69 years	1022	8.4 (0.5)		39.6 (7.2)		1.7 (0.5)	
Social class I	2793	8.0 (0.4)	<0.001	32.9 (7.0)	<0.001	1.3 (0.4)	<0.001
Social class II	13,255	8.1 (0.5)		33.7 (7.2)		1.4 (0.4)	
Social class III	23,833	8.1 (0.5)		36.3 (8.1)		1.5 (0.5)	
Smokers	13,040	8.1 (0.5)	<0.001	35.6 (8.0)	<0.001	1.5 (0.5)	<0.001
Non Smokers	26,841	8.0 (0.5)		34.3 (7.6)		1.4 (0.5)	
Yes Mediterranean diet	20,344	7.9 (0.4)	<0.001	30.6 (3.7)	<0.001	1.2 (0.2)	<0.001
Non Mediterranean diet	19,537	8.3 (0.5)		40.0 (8.2)		1.8 (0.5)	
Yes physical activity	20,669	7.9 (0.4)	<0.001	30.2 (3.5)	<0.001	1.2 (0.2)	<0.001
Non physical activity	19,212	8.3 (0.5)		40.5 (7.8)		1.8 (0.5)	
SF-12 good	32,173	8.0 (0.4)	<0.001	33.1 (6.0)	<0.001	1.3 (0.3)	<0.001
SF-12 poor	7708	8.5 (0.5)		44.0 (8.8)		2.0 (0.6)	

SF-12: Short Form Health Survey. TyG: Triglyceride-Glucose Index. METS-Ir: metabolic score for insulin resistance. SPISE-IR: Single Point Insulin Sensitivity. SD: standard deviation.

**Table 3:** Prevalence of Elevated TyG, METS-IR, and SPISE Scores by Sociodemographic and Lifestyle Factors, Stratified by Sex.

		**TyG index High**		**METS-IR High**		**SPISE-IR High**	
**Men**	***n***	**%**	***p*** **-Value**	**%**	***p*** **-Value**	**%**	***p*** **-Value**
18–29 years	10,774	10.0	<0.001	3.4	<0.001	5.7	<0.001
30–39 years	19,795	20.0		6.4		10.6	
40–49 years	17,850	30.6		10.1		16.9	
50–59 years	9915	35.1		13.3		20.2	
60–69 years	1799	35.3		13.4		20.5	
Social class I	3208	20.7	<0.001	6.7	<0.001	11.3	<0.001
Social class II	10,602	24.0		7.5		12.3	
Social class III	46,323	24.6		8.6		13.9	
Smokers	22,265	27.5	<0.001	7.9	<0.001	13.8	<0.001
Non-smokers	37,868	22.4		9.0		13.2	
Yes Mediterranean diet	24,790	2.3	<0.001	4.1	<0.001	5.9	<0.001
Non Mediterranean diet	35,343	39.7		10.2		14.3	
Yes physical activity	27,551	1.9	<0.001	3.3	<0.001	4.4	<0.001
Non physical activity	32,582	43.2		12.2		16.2	
SF-12 good	41,843	8.2	<0.001	4.0	<0.001	5.5	<0.001
SF-12 poor	18,290	61.2		10.5		16.1	
Women	*n*	%	*p*-value	%	*p*-value	%	*p*-value
18–29 years	7747	6.0	<0.001	3.5	<0.001	4.7	<0.001
30–39 years	13,365	7.3		4.7		6.6	
40–49 years	11,626	12.5		6.6		8.9	
50–59 years	6121	20.6		8.1		11.6	
60–69 years	1022	26.2		10.0		15.4	
Social class I	2793	6.9	<0.001	3.4	<0.001	4.7	<0.001
Social class II	13,255	9.6		4.0		5.5	
Social class III	23,833	12.4		6.9		9.6	
Smokers	13,040	11.9	<0.001	6.2	<0.001	8.5	<0.001
Non Smokers	26,841	10.6		4.6		6.7	
Yes Mediterranean diet	20,344	5.1	<0.001	3.8	<0.001	4.9	<0.001
Non Mediterranean diet	19,537	14.3		8.7		10.2	
Yes physical activity	20,669	3.8	<0.001	3.1	<0.001	4.1	<0.001
Non physical activity	19,212	16.9		10.4		12.6	
SF-12 good	32,173	6.5	<0.001	4.2	<0.001	5.0	<0.001
SF-12 poor	7708	22.3		8.0		10.1	

SF-12 Short Form Health Survey. TyG Triglyceride-Glucose index. METS-Ir Metabolic score for insulin resistance. SPISE-IR Single Point Insulin Sensitivity.

**Table 4:** Multivariate Logistic Regression Analyses of Factors Associated with High TyG, METS-IR, and SPISE-IR Scores.

	**TyG index High**		**METS-IR High**		**SPISE-IR High**	
	**OR (95% CI)**	***p*** **-Value**	**OR (95% CI)**	***p*** **-Value**	**OR (95% CI)**	***p*** **-Value**
Women	1		1		1	
Men	2.35 (2.25–2.46)	<0.001	1.85 (1.70–2.01)	<0.001	1.26 (1.20–1.33)	<0.001
18–29 years	1		1		1	
30–39 years	1.12 (1.10–1.15)	<0.001	1.19 (1.14–1.24)	<0.001	1.15 (1.10–1.21)	<0.001
40–49 years	1.29 (1.24–1.34)	<0.001	1.42 (1.30–1.55)	<0.001	1.34 (1.25–1.44)	<0.001
50–59 years	1.41 (1.35–1.47)	<0.001	2.08 (1.70–2.46)	<0.001	1.59 (1.47–1.72)	<0.001
60–69 years	1.60 (1.50–1.71)	<0.001	3.11 (2.51–3.72)	<0.001	1.88 (1.69–2.08)	<0.001
Social class I	1		1		1	
Social class II	1.15 (1.12–1.18)	<0.001	1,15 (1.10–1.21)	<0.001	1.19 (1.13 -1.25)	<0.001
Social class III	1.44 (1.37–1.52)	<0.001	1.43 (1.35–1.52)	<0.001	1.42 (1.32–1.53)	<0.001
Non smokers	1		1		1	
Smokers	1.50 (1.41–1.60)	<0.001	1.14 (1.10–1.19)	<0.001	1.21 (1.16–1.27)	<0.001
Yes Mediterranean diet	1		1		1	
Non Mediterranean diet	2.13 (1.85–2.41)	<0.001	2.66 (2.17–3.16)	<0.001	2.78 (2.40–3.17)	<0.001
Yes physical activity	1		1		1	
Non physical activity	5.39 (4.50–6.29)	<0.001	6.23 (5.10–7.36)	<0.001	6.67 (5.39–7.96)	<0.001
SF-12 good	1		1		1	
SF-12 poor	3.83 (3.23–4.24)	<0.001	3.29 (2.67–3.92)	<0.001	4.11 (3.20–5.01)	<0.001

## Data Availability

All data generated or analyzed in this study are securely archived at ADEMA University School in compliance with applicable data protection laws. Oversight is provided by the institution’s Data Protection Officer, Mr. Ángel Arturo López González. Due to confidentiality agreements, data are not publicly available but may be provided upon reasonable request and with appropriate ethical approval.

## Supplementary Materials

The following supporting information can be downloaded at: https://www.mdpi.com/article/10.3390/medsci13030122/s1. Table S1. Correlations Between Individual SF-12 Items and Insulin Resistance Indices (TyG, METS-IR, SPISE-IR) in Workers.

## Appendix A

### Appendix A.1.1. SF-12 Health Survey (English Version)

Instructions to Respondent:

The following questions ask for your views about your health. This information will help keep track of how you feel and how well you are able to do your usual activities.

1. In general, would you say your health is:

( ) Excellent ( ) Very good ( ) Good ( ) Fair ( ) Poor

2. Compared to one year ago, how would you rate your health in general now?

( ) Much better now ( ) Somewhat better ( ) About the same ( ) Somewhat worse ( ) Much worse

3. The following questions are about activities you might do during a typical day. Does your health now limit you in these activities? If so, how much?
  - Moderate activities (e.g., moving a table, vacuuming, bowling, or playing golf)
  ( ) Yes, limited a lot ( ) Yes, limited a little ( ) No, not limited at all
  b. Climbing several flights of stairs
  ( ) Yes, limited a lot ( ) Yes, limited a little ( ) No, not limited at all
4. During the past 4 weeks, have you had any of the following problems with your work or other regular daily activities as a result of your physical health?
  - Accomplished less than you would like
  ( ) Yes ( ) No
  b. Were limited in the kind of work or other activities
  ( ) Yes ( ) No
5. During the past 4 weeks, have you had any of the following problems with your work or other regular daily activities as a result of any emotional problems (such as feeling depressed or anxious)?
  - Accomplished less than you would like
  ( ) Yes ( ) No
  b. Didn’t do work or other activities as carefully as usual
  ( ) Yes ( ) No
6. During the past 4 weeks, how much did pain interfere with your normal work (including both work outside the home and housework)?
  ( ) Not at all ( ) A little bit ( ) Moderately ( ) Quite a bit ( ) Extremely
7. These questions are about how you feel and how things have been with you during the past 4 weeks.
  - Have you felt calm and peaceful?
  - Did you have a lot of energy?
  - Have you felt downhearted and blue?

Response options for each:

( ) All of the time ( ) Most of the time ( ) A good bit of the time

( ) Some of the time ( ) A little of the time ( ) None of the time

8. During the past 4 weeks, how much of the time has your physical health or emotional problems interfered with your social activities (like visiting with friends, relatives, etc.)?

( ) All of the time ( ) Most of the time ( ) Some of the time ( ) A little of the time ( ) None of the time

### Appendix A.1.2. Scoring and Interpretation

The SF-12 is scored using a norm-based method, which calculates two summary measures:

- Physical Component Summary (PCS)
- Mental Component Summary (MCS)

Each item response is recoded and weighted according to algorithms developed from general population norms. Scores are standardized with a mean of 50 and a standard deviation of 10.

Interpretation:

- Below 50: Below average health status
- 50: Average health status
- Above 50: Better than average health status
