# Supplementary material for: Sociodemographic Factors, Healthy Habits, and Quality of Life in Relation to Insulin Resistance Risk in a Large Cohort of Spanish Workers
Source: Med Sci (Basel). 2025 Aug 11;13(3):122. doi: 10.3390/medsci13030122 (PMC12371977; doi:10.3390/medsci13030122)
Supplement: Supplementary file 1 [file medsci-13-00122-s001.zip › medsci-3812963-supplementary.pdf]

Table S1. Correlations Between Individual SF-12 Items and Insulin Resistance Indices (TyG, METS-IR, SPISE-IR) in Workers.

SF-12 Item	TyG index	METS-IR	SPISE-IR
General Health	0.0447	-0.0256	0.0346
Physical Functioning	-0.0187	-0.0571	-0.0724
Role Physical	0.0067	-0.0261	-0.0328
Bodily Pain	-0.0465	-0.0106	0.0198
Vitality	0.0144	-0.0997	-0.0031
Social Functioning	0.0126	-0.0468	0.0564
Role Emotional	-0.0573	0.0294	-0.0465
Mental Health	0.0158	0.0045	0.0280
